# Whole-exome sequencing identifies Y1495X of *SCN5A* to be associated with familial conduction disease and sudden death

**DOI:** 10.1038/srep05616

**Published:** 2014-07-10

**Authors:** Zhi-Ping Tan, Li Xie, Yao Deng, Jin-Lan Chen, Wei-Zhi Zhang, Jian Wang, Jin-Fu Yang, Yi-Feng Yang

**Affiliations:** 1 Department of Cardiothoracic Surgery, the Second Xiangya Hospital of Central South University, Changsha, Hunan Province 410011, China; 2 Clinical Center for Gene Diagnosis and Therapy of State Key Laboratory of Medical Genetics, the Second Xiangya Hospital of Central South University, Changsha, Hunan Province 410011, China

## Abstract

*SCN5A* mutations have been reported to underlie a variety of inherited arrhythmias, while the complex overlapping phenotype, especially with congenital heart disease (CHD), is rarely reported. The 48-year-old proband underwent a recent syncope during rest. A CHD (tetralogy of Fallot) and conduction disease was revealed by echocardiogram and ultrasonic cardiogram examination. We combined whole-exome sequencing (WES) and bioinformatics strategies to identify the pathogenic gene for this autosomal-dominant cardiac conduction disease (CCD) in a multi-generation pedigree. We examined four members of this family, including three affected and one unaffected. A novel nonsense mutation (Y1495X) in *SCN5A* was identified in the affected family members. This mutation is predicted to generate a truncated SCN5A protein, which could result in the loss of sodium current, a defined mechanism of *SCN5A* related arrhythmias. Our study provides evidence that WES is a highly effective approach for genetic analyses of rare clinical phenotypes. Our study also offers accurate genetic testing information for those yet clinically negative relatives.

Cardiac conduction disease is a serious disorder and the leading cause of mortality worldwide^1^. Conduction slowing of the electric impulse may result in syncope and sudden cardiac death (SCD)^2^,^3^. The human voltage-gated cardiac sodium channel generates the initial fast upstroke of the action potentials. Mutations in *SCN5A*, the gene that encodes the pore-forming subunit of human cardiac sodium channel Nav1.5, have been associated with a variety of inherited arrhythmogenic syndromes including type 3 long-QT syndrome (LQT3)^4^, Brugada syndrome (BrS)^5^, progressive cardiac conduction disease (PCCD)^6^, sick sinus syndrome (SSS)^7^, atrial fibrillation (AF)^8^, dilated cardiomyopathy (DCM)^9^ and more complex overlapping syndrome^10^,^11^.

There is a rapid growing interest for clinical diagnosis of the disease genes that underlie genetic heart rhythm disorders since the discovery of the first Long QT type 3 syndrome-associated genes in 1995^4^,^12^, As mentioned, a collection of cardiac diseases are caused by a single channelopathy gene *SCN5A*. Alternatively, 13 distinct disease-causing genes are implicated in a single OMIM entity, the conduction system disease^13^, suggesting a more complex genetic heterogeneity than previously thought. The high genetic heterogeneity, variable expressions, reduced penetrance and pleiotropic effects of arrhythmias-related genes have impeded the identification of pathogenic gene^14^,^15^,^16^, especially for channelopathies with rare clinical phenotypes.

In the present study, we investigated a clinically characterized family with a history of CCD, syncope and sudden cardiac death. An obvious autosomal-dominant inheritance with discordant cardiac abnormalities (e.g. conduction disease, congenital heart disease and bradyarrhythmias) has been observed in this family. Since congenital heart diseases (CHD), especially tetrology of Fallot (TOF), have been rarely reported in channelopathies, we hypothesize that this family might represent a new variant or an uncharacterized inherited cardiac channelopathy. By whole-exome sequencing of four family members (three affected and one normal control), we identified a novel nonsense mutation Y1495X in *SCN5A* that might underlie this unique inherited cardiac arrhythmia.

## Methods

### Patients and subjects

The study protocol was approved by the Review Board of the Second Xiangya Hospital of the Central South University in China, and the study participants gave informed consent. All experiments were performed in accordance with relevant guidelines and regulations. We enrolled 37 members of this family (10 affected, 25 unaffected and 2 unknown) in the study. Blood were obtained from the affected probands and family members. Subjects were reviewed by medical records including 12-lead echocardiogram (ECG), ultrasonic cardiogram (UCG) and hospital records. PR, QRS interval, QT, QTc duration and QRS axis were measured. The normal range of the ECG measurements was based on the age of each individual. For adult, a QRS interval > 100 ms and a PR interval > 210 ms were considered prolonged^17^.

### DNA extraction

Genomic DNA was extracted from peripheral blood lymphocytes of the family members. Genomic DNA was prepared using a DNeasy Blood & Tissue Kit (Qiagen, Valencia, CA) on the QIAcube automated DNA extraction robot (Qiagen, Hilden, Germany) as previously described^18^.

### Targeted Capture and Massive Parallel Sequencing

Exome capture and high-throughput sequencing (HTS) were performed in the State Key Laboratory of Medical Genetics of China (SKLMG) in collaboration with Beijing Genomics Institute (BGI Shenzhen)^19^. Five micrograms of genomic DNA from 4 family members (three affected, II-3, III-10, IV-1 and one normal control, III-14) were captured with the NimbleGen SeqCap EZ library exome capture reagent (Roche Inc., Madison, USA) and sequenced (Illumina Hiseq2000, 90 base-paired end reads) (Illumina Inc, San Diego, USA). Briefly, genomic DNA was randomly fragmented by Covaris S2 instrument (Covaris, Inc., Woburn, USA). Then the 250–300 bp fragments of DNAs were subjected to three enzymatic steps: end repair, A-tailing and adapters ligation. Once the DNA libraries were indexed, they were amplified by ligation-mediated PCR. Extracted DNA was purified and hybridized to the NimbleGen SeqCap EZ Library. Each captured library was then loaded to the Illumina Hiseq2000 platform. Illumina base calling software v1.7 was employed to analyze the raw image files with default parameters.

### Reads, Mapping and Variant detection

SNP analysis was performed as previously described^20^: (i) Reads were aligned to the NCBI human reference genome (gh19/NCBI37.1) with SOAPaligner method v2.21; (ii) for paired-end reads with duplicated start and end sites, only one copy with the highest quality was retained, and the reads with adapters were removed; (iii) SOAPsnp v1.05 was used to assemble the consensus sequence and call genotypes;(iv) small indel detection was used with the Unified Genotyper tool from GATK v1.0.4705.

### Filtering and Annotation

Four major steps were taken to prioritize all the high-quality variants^20^: (a) variants within intergenic, intronic, and UTR regions and synonymous mutations were excluded from later analysis; (b) variants in dbSNP132 (http://www.ncbi.nlm.nih.gov/projects/SNP/), the 1000 Genomes project (1000G,www.1000genomes.org), and HapMap Project (ftp://ftp.ncbi.nlm.nih.gov/hapmap) were excluded; (c) Variants in YH Database (http://yh.genomics.org.cn/) and NHLBI Exome Sequencing Project (ESP) database (http://evs.gs.washington.edu/EVS/) were further excluded; (d) SIFT (http://sift.bii.astar.edu.sg/), Polyphen2 (http://genetics.bwh.harvard.edu/pph2/), MutationTaster (www.mutationtaster.org) and GERP (UCSC Genome Browser) were used to predict the possible impacts of variants.

### Mutation Validation and Co-segregation Analysis

Sanger sequencing was used to validate the candidate variants found in whole-exome sequencing. Segregation analyses were performed in the family members. Primers pairs used to amplify fragments encompassing individual variants were designed using an online tool (PrimerQuest, IDT) (http://www.idtdna.com/Primerquest/Home/Index), and the sequences of the primers are listed in Supplementary Table S1.

## Results

### Clinical features

**Figure 1:**
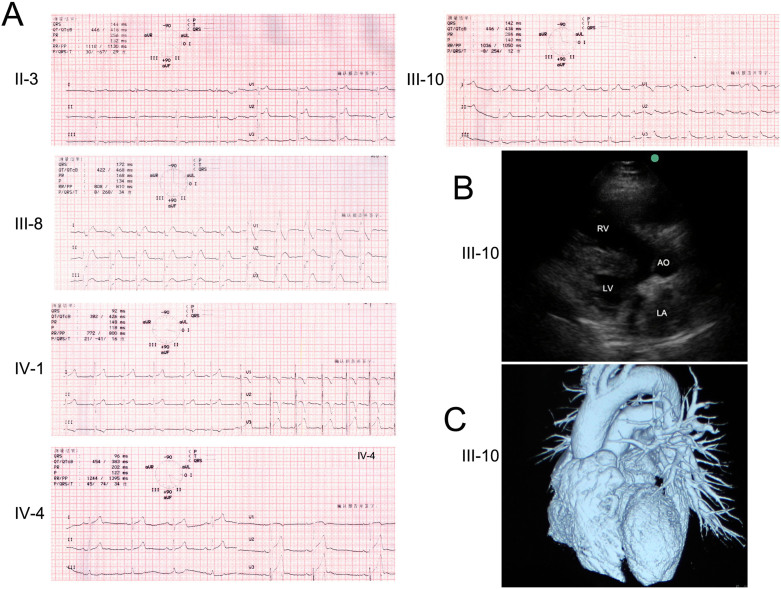
Clinical features of family members with conduction diseases. (A) Electrocardiograms (ECGs) of the family members (II-3, III-8, IV-1, IV-4) and the proband (III-10). (B) Ultrasonic cardiogram (UCG) of proband shows cardiac structural defect, tetralogy of Fallot (TOF). (C) Computed tomography (CT) of the proband's heart shows a pulmonary hypoplasia.

We identified a Chinese family with multiple complex phenotypes including conduction diseases, CHD and sudden cardiac death (Fig. 1A). The proband (III-10), a 48-year-old farmer from Hunan province of Central-South China, had a syncope during rest after hard manual labor. He had no record of symptom for almost 40 years since his first syncope at seven years old. Physical examination showed a congenital heart disease (tetrology of Fallot, TOF) and cardiac conduction disease (Fig. 1B, C). Family history examination showed his mother died at 35 years old during sleep for unknown reason. One of the proband's maternal uncles also died during sleep at 37 years old. Due to a high risk of surgical correction for adult TOF and conduction disease, the proband was discharged without surgery. Currently, he is in a stable condition.

Eight family members, along with the proband, had a history of syncope. We recruited all family members for further physical examination with ECG and UCG. Family member II-3, II-8, III-8, III-10, III-18 and IV-1 had a prolonged QRS without CHD (Table 1). The son of the proband (IV-4) had sinus bradycardia with a heart rate of 37 (beats/min) and a prolonged PR interval (202 ms) (Table 1).

### Genetic analysis

**Figure 2:**
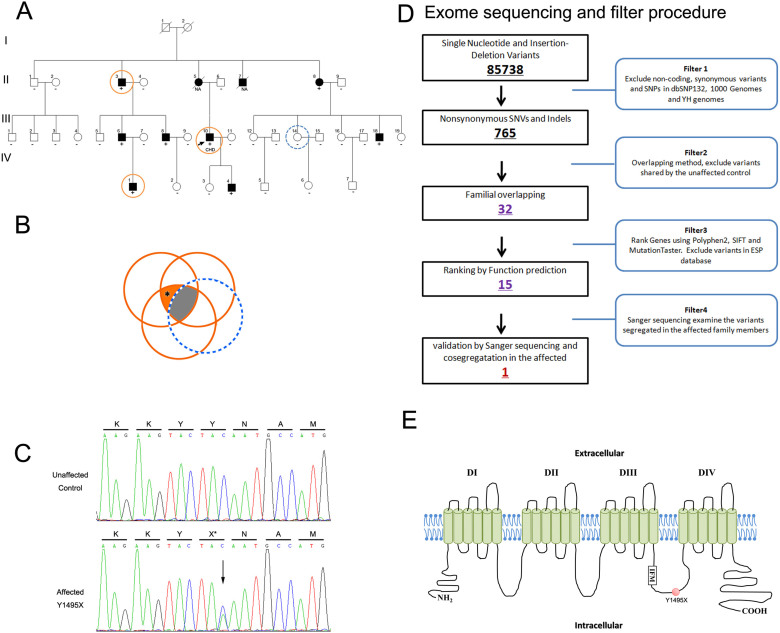
Whole exome sequencing and familial genomic tetra-overlapping for the identification of a novel genetic variant for multiple complex syndromes. (A) Pedigree of the family. Black circles/squares are affected, white are unaffected. Arrow indicates the proband. 4 large circles (3 in red and 1 in blue) represent the 4 individuals underwent whole exome sequencing. Plus signs indicate Y1495 *SCN5A* mutation positive. Minus signs indicate mutation-negative. NA represents DNA samples were not available. (B) Overlapping filter strategy. Asterisks denotes remaining variants for further analysis that are present in 3 affected members (red circles) but not in the normal control (blue circle). (C) Sanger DNA sequencing chromatogram demonstrates the heterozygosity for a *SCN5A* mutation (p.Y1495X, c.4485C > A). (D) Schematic representation of the filter strategies employed in our study.

Because CHD is rarely reported in arrhythmias, we postulated that this pedigree may represent a rare undescribed inherited cardiac channelopathy. To identify the underlying genetic cause, genomic DNA samples of four participants of the family, including the proband (III-10), his affected uncle II-3, the grandson of the affected uncle (IV-4) and his unaffected maternal sister (III-14), were analyzed by WES (Fig. 2A). For the four samples, WES yielded an average of 10 Gb data with an appropriately 95% coverage of target region and a 93% of target covered over 10× (Table 2).

The detected SNV and INDELs were analyzed by multiple filter strategies. After alignment and SNV calling, 85,738 variants were present in the exome of the proband (Table 2). After exclusion of shared common variants present in dbSNP132, 1000 Genomes Project and BGI in-house control (2500 exomes) as well as ESP database, 765 unique SNPs were identified (Table 2). Variants shared by three affected family members (II-3, III-10 and IV-1) but not present in the unaffected normal control (III-14) were identified, in which 32 rare variants were further analyzed (Supplementary Table S2).

Of the 32 variants, 15 were ranked using three different bioinformatic programs (Supplementary Table S3) and chosen as the candidates for pathogenicity of this unique family. Sanger DNA sequencing was employed to examine the mutation segregated with affected members (PCR primers are listed in Supplementary Table S3), which indicates that a novel nonsense 1495× mutation in *SCN5A* as the underlying genetic lesion of these patients (Fig. 2).

To explore the etiology of CHD in the proband by known genetic mutation, we focused on the 765 remaining variants of the proband and revised our strategy with a filter of 456 CHD-related genes (Supplementary Table S4). The single patient analysis excluded the possibility of a known causative gene that underlie CHD.

## Discussion

In this study, we employed whole-exome sequencing to explore the possible causative gene for a family with overlap syndrome including CHD, conduction diseases and sudden cardiac death and identified a novel nonsense mutation in codon 1495 of *SCN5A*. The mutation locates in the highly conserved triad IFM motif (formed by residues 1485–1487)^21^ and is predicted to result in a premature protein truncation that is closely associated with channelopathies. Our study is consistent with previous reports that nonsense mutations cause more severe phenotypes in humans as well as animal models^22^,^23^,^24^,^25^.

Whole-exome sequencing (WES) has developed into a powerful and cost-effective tool to uncover genetic basis for rare Mendelian diseases^26^,^27^. WES also shows tremendous potential in genetic diagnostics that helps to expand the clinical spectrum of known diseases^28^,^29^,^30^. However, one of the major challenges in WES is to discriminate the pathogenic variants from the benign ones^31^,^32^. Several strategies with different filters have been developed to exclude variants that are unlikely to cause disease. Recently WES coupling with bioinformatics method in cardiovascular genetic study leads to the identification of *CACNA1C* in Long QT syndrome^33^. Based on a similar strategy, we revised the filter strategy to a tetra-overlapping filer, which resulted in a significant decrease of candidate genes (32 genes) in our study and is an obvious improvement over the 110 candidate genes of the triangulation strategy in the aforementioned study^33^.

The cause of the CHD in the proband of the family is unclear. Channelopathies (LQT3 and BS) are often found with a structurally normal heart. However there have been a few reports about the links between ion channel gene *SCN5A* mutation and CHD^15^. In 2002, Bezzina *et al* reported a small muscular ventricular septal defect (VSD) in the index patient of a familial conduction disease harboring co-occurrence mutations (W156X and R225W)^34^. Recently, an Ebstein's anomaly was reported in a German boy with severe conduction disease carrying a homozygous mutation (I230T)^35^. CHDs are also observed in individuals carrying mutations in other channelopathy disease-causing genes. For example, Timothy Syndrome (LQT8, mutated in *CACNA1C*) is reported to have multiple anomalies including congenital heart diseases (e.g. tetralogy of Fallot)^36^. We have excluded known CHD genes as the underlying genetic causes. However it remains a possibility, though rare, that the CHD phenotype (TOF) is caused by an unidentified genetic mutation as described by “the second hit model”^37^,^38^.

Our result suggests that the nonsense mutation (Y1495X) in *SCN5A* might be the causal genetic lesion of multiple overlap syndromes including conduction disease, sudden death and CHD in a single family, reinforcing the previously speculated mechanistic link between cardiac channelopathies and overlap syndromes^38^,^39^. In addition, the CHD patient (TOF) carrying this mutation may offer a valuable chance to examine the relationship between TGF-β1 mediated fibrosis, aging processes and *SCN5A* in human heart, a possibility suggested by recent findings in Scn5a knockout animal models^23^,^40^,^41^,^42^. Since the correction of TOF needs an open-heart surgery, we might be able to observe the exact evidence during the procedure of the surgery, likely an unprecedented study for channelopathy diseases. Open-heart surgery of TOF usually requires excision of large amount of cardiac papillary muscle in order to discharge the right ventricular outflow tract obstruction (RVOTs), which might offer an exciting alternative for cellular electrophysiological study with primary cardiac cells, an experiment currently performed only with induced pluripotent stem cell-derived (iPS) cardiomyocytes^43^,^44^.

## Conclusions

Using whole-exome sequencing in combination with bioinformatics analyses, we identified the nonsense mutation (Y1495X) in *SCN5A* as a possible cause of overlap syndrome including CHD, conduction diseases and sudden cardiac death in a Chinese family. Our study suggests that this approach will facilitate our understanding of the etiology of this rare type of arrhythmias by effective identification of the causative gene mutations and should promote the diagnosis and treatment of these diseases.

## Supplementary Material

Supplementary Information Table S1

Supplementary Information Table S2

Supplementary Information Table S4

Supplementary Information Table S3

## Figures and Tables

**Table 1:** Demographic and electrocardiographic features in members of the family

							ECG intervals (ms)				
Member	Sex	Age (Years)	Symptoms	Clinical ECG Diagnosisi	QRS Axis	Heart Rate (Beats/min)	PR	QRS	QT/QTc	others	Mutation (Y1495X)
II-1	M	76	Asymptomatic	no	−2	83	190(210)	82(100)	368/432		-
II-3*	M	72	Syncope(>20)	Sinus bradycardia, LAFB, T wave abnormalities	−67	**50**	**256**(210)	**144**(100)	446/418		+
II-5	F	35 †	Syncope	NA	NA	NA	NA	NA	NA	Sudden death	NA
II-7	M	37 †	Syncope	NA	NA	NA	NA	NA	NA	Sudden death	NA
II-8	F	64	Syncope(>10)	RBBB	−65	**52**	**244**(210)	**140**(100)	438/426		+
III-6	M	39	3syncopes	RBBB		61	208(210)	**134**(100)	430/432		+
III-8	M	35	3 syncopes	RBBB	268	74	168(210)	**172**(100)	422/468		+
III-10*#	M	48	3 syncopes	Sinus bradycardia, first degree AVB	254	**54**	**284**(210)	**142**(100)	446/434	CHD (TOF)	+
III-14*	F	46	Asymptomatic	no	42	82	138(210)	80(90)	370/432		−
III-18	M	30	2 syncopes	RBBB	14	56	204(210)	**138**(100)	434/434		+
IV-1*	M	8	1 syncope	LAFB	−41	75	148(170)	**92**(90)	382/426		+
IV-2	F	5	Asymptomatic	no	42	124	128(170)	62(90)	306/439		−
IV-4	M	18	2 syncopes	Sinus bradycardia	74	**37**	**202**(180)	96(100)	454/383		+

NA = not available; QTc = corrected QT interval; RBBB = Right Bundle Branch Block; LAFB = Left anterior fascicular block. AVB = atrioventricular Block; CHD = congenital heart disease; TOF = tetralogy of Fallot.

+ Y1495X present, − Y1495X absent.

*Whole- exome sequenced.

^†^Age of death.

#Index case.

**Table 2:** Summary of SNPs for exome captured samples and filter procedure

Categories	III-10*	II-3	IV-1	III-14 (control)
**Exome Capture Statistics**				
Target region (bp)(1)	62085286	61988644	62085286	62085286
Raw reads	92737976	138497858	145426084	139851618
Raw data yield (Mb)	9465	11149	11707	11258
Reads mapped to genome	93894979	140334153	147846058	142256214
Reads mapped to target region(2)	53479844	82582631	87924642	82990685
Data mapped to target region (Mb)	3677.14	5658.73	6019.33	5690.76
Coverage of target region (%)	95.68	95.93	95.69	95.84
Average read length (bp)	80.5	80.5	80.51	80.5
Fraction of target covered > = 10X (%)	93.27	94.02	93.93	93.96
Fraction of target covered > = 20X (%)	89.6	91.86	91.83	91.76
Fraction of target covered > = 30X (%)	81.92	88.12	88.16	87.88
**SNPs for exome capture**				
No. high-confidence genotypes	115744429	115676179	113537889	114980456
Total number of SNPs	85738	91309	89061	89972
Missense	11032	11352	11127	11269
Nonsense	127	144	143	143
Splice site(3)	2498	2675	2651	2682
Synonymous-coding	9600	9934	9792	9894
Hom	36615	37234	37483	37668
Het	49123	54075	51578	52304
Novel variants				
Number not in dbSNP	1287	1373	1358	1241
Number not in 1000 Genome and YH	765	787	779	755
Missense	624	631	632	610
Nonsense	15	21	23	19
Splice site	126	135	124	126
No. of genes presented in 3 affected patients but not in the normal family member III-14				32
Top-ranked detrimental effect of variants by three distinct programs (Polyphen2, SIFT and MutationTaster)				15
No. of validated variants co-segregating with affected members				1

*Index patient.

(1)Target regions refer to the regions that are actually covered by the designed probes.

(2)Reads mapped to target regions are reads that within or overlap with target region.

(3)Intronic SNPs within 10 bp of exon/intron boundary.
