# Supplementary material for: Whole-exome sequencing identifies Y1495X of SCN5A to be associated with familial conduction disease and sudden death
Source: Sci Rep. 2014 Jul 10;4:5616. doi: 10.1038/srep05616 (PMC5375973; doi:10.1038/srep05616)
Supplement: Supplementary Information — Table S4 [file srep05616-s3.doc]

**Whole-exome sequencing identifies Y1495X of *SCN5A* to be associated with familial conduction disease and sudden death**

Zhi-Ping Tan^1, 2 *^, Li Xie^1^, Yao Deng^1^, Jin-Lan Chen^1^, Wei-Zhi Zhang^1^, Jian Wang^1, 2^, Jin-Fu Yang^1, 2^, and Yi-Feng Yang^1, 2, *^

1. Department of Cardiothoracic Surgery, the Second Xiangya Hospital of Central South University, Changsha, Hunan Province 410011, China.

2. Clinical Center for Gene Diagnosis and Therapy of State Key Laboratory of Medical Genetics, the Second Xiangya Hospital of Central South University, Changsha, Hunan Province 410011, China.

Supplementary Table S4 456 CHD-related genes for filter [^1-5^](#_ENREF_1)

| ABCC9 | ACE | ACP6 | ACTA2 | ACTB | ACTC1 | ACTN2 | ACVR1 | ACVR2B | ADRB1 |
| --- | --- | --- | --- | --- | --- | --- | --- | --- | --- |
| ADRB2 | ADRB3 | AGL | AGT | AGTR1 | AHSA2 | ANKRD1 | APOBEC2 | ARL13B | ASXL2 |
| ATE1 | ATP1A2 | ATP4A | ATP4B | BAT1 | BBIP1 | BBS1 | BBS10 | BBS12 | BBS2 |
| BBS4 | BBS5 | BBS7 | BBS9 | BCL11A | BCL6 | BCL9 | BCOR | BICC1 | BMP7 |
| BMPR1A | BMPR1B | BMPR2 | BUB1B | C1ORF106 | CACNA1C | CACNA2D1 | CALM1 | CALM2 | CALR3 |
| CASQ2 | CAV3 | CCDC39 | CCDC40 | CCT4 | CDH1 | CDH2 | CDKN1C | CER1 | CFC1 |
| CHD1L | CHD7 | LDB3 | FH19 | APPB1 | FIBP | KMT2D | GPD1 | TTC8 | NGF |
| HMGCL | STIL | MKKS | RPSA | ARL6 | NELFA | GRID2 | KCNH2 | TRIM32 | CHRAC1 |
| CHRD | CHRNG | PQBP1 | CITED2 | CLDN7 | CLUL1 | CNTF | COL11A1 | COL11A2 | COL2A1 |
| COL3A1 | CREBBP | CRELD1 | CRHBP | CRX | CRYAB | CSRP1 | CSRP3 | CTLA4 | CTNNA3 |
| CUL3 | CYP11B2 | DAND5 | DAPK3 | DES | DHCR24 | DHCR7 | DHODH | DLL1 | DMRT2 |
| DNAI1 | DNAI2 | DOLK | DOT1L | DPP6 | DPPA4 | DSC2 | DSG2 | DSP | DST |
| DTNA | DVL1 | DVL2 | DZIP1 | EDNRA | EDNRB | EED | EFNB1 | EHMT1 | ELN |
| EMD | EP300 | ESCO2 | EVC | EVC2 | EYA4 | EZH1 | EZH2 | FBN1 | FBN2 |
| FGB | FKTN | FLNA | FLNB | FMO5 | FOXA2 | FOXC1 | FOXC2 | FOXH1 | FOXJ1 |
| FOXL2 | FTO | FXN | GAA | GADL1 | GALNT11 | GATA4 | GATA5 | GATA6 | GATAD1 |
| GDF1 | GJA1 | GJA5 | GJA8 | GJA9 | GLA | GLI2 | GLI3 | GPC3 | GPD1L |
| GPR161 | GPRC6A | GSK3B | HAND1 | HAND2 | HCN4 | HES1 | HES4 | HEY2 | HFE |
| HOXA1 | HUWE1 | HYLS1 | ID2 | IDUA | IER2 | IFNG | IFT122 | IFT172 | IFT20 |
| IFT57 | IFT88 | IGFBP4 | IGFBP5 | IHH | IL10 | IPPK | ISL1 | JAG1 | JARID2 |
| JAZF1 | JPH2 | JUP | KCND2 | KCND3 | KCNE1 | KCNE1L | KCNE2 | KCNE3 | KCNE4 |
| KCNH2 | KCNJ11 | KCNJ2 | KCNJ5 | KCNJ8 | KCNMB1 | KCNQ1 | KDM5A | KDM5B | KDM6A |
| KIAA0196 | KIAA1841 | KIF3A | KIF3B | KIF3C | KIFAP3 | KLF13 | KRAS | LAMA4 | LAMP2 |
| LBR | LDB3 | LEFTY1 | LEFTY2 | LEMD3 | LIPC | LLPH | LMNA | LPIN1 | LRRC50 |
| LRRC6 | MARK2 | MAX | MED13L | MED20 | MEF2A | MEF2C | METT10D | MGAT1 | MGP |
| MICA | MICB | MID1 | MKKS | MKRN2 | MKS1 | MNDA | MSX2 | MYBPC3 | MYH10 |
| MYH11 | MYH6 | MYH7 | MYL2 | MYL3 | MYLK2 | MYOZ2 | MYPN | NAA15 | NCOR2 |
| NEBL | NEK2 | NEXN | NF1 | NFATC1 | NFATC3 | NFATC4 | NFKBIL1 | NIPBL | NKD1 |
| NKX2-5 | NKX2-6 | NKX3-2 | NODAL | NOS3 | NOTCH1 | NOTCH2 | NOTCH2NL | NOTCH3 | NOTCH4 |
| NOTO | NPHP3 | NPPA | NPPB | NSD1 | NUB1 | NUMBL | NUP188 | OBSCN | OFD1 |
| OSR1 | PAFAH1B1 | PAPOLG | PCMTD2 | PCSK5 | PDLIM3 | PEX1 | PEX13 | PHF8 | PHYHD1 |
| PIFO | PITX2 | PKD1L1 | PKD2 | PKP2 | PLA2G7 | PLAGL1 | PLN | PPM1K | PPP3CA |
| PQBP1 | PRC1 | PRDM1 | PRKAB2 | PRKAG2 | PROX1 | PSEN1 | PSEN2 | PTCH1 | PTCH2 |
| PTPLA | PTPN11 | PTPN22 | PTPRC | RAB10 | RAB23 | RAF1 | RAI1 | RAI2 | RANGRF |
| RAPGEF5 | RBM20 | REL | RFX2 | RFX3 | RIT1 | RNF20 | ROCK2 | ROR2 | RPGRIP1L |
| RUNX2 | S100Z | SALL1 | SALL2 | SALL4 | SATB2 | SCN1B | SCN3B | SCN4B | SCN5A |
| SDC2 | SDHA | SEL1L3 | SEMA3E | SESN1 | SETBP1 | SGCA | SGCB | SGCD | SGCE |
| SGCG | SHH | SHOC2 | SIX3 | SLC26A2 | SLC2A10 | SLMAP | SMAD2 | SMAD5 | SMARCD3 |
| SMO | SMYD1 | SMYD2 | SNAI1 | SNTA1 | SOD2 | SOS1 | SOX17 | SOX9 | SRF |
| STIL | SUFU | SUPT3H | SUPT5H | SUV420H1 | TAZ | TBX1 | TBX20 | TBX3 | TBX5 |
| TCAP | TCF21 | TCOF1 | TDGF1 | TFAP2A | TFAP2B | TGFB1 | TGFBR1 | TGFBR2 | TGIF1 |
| TLL1 | TMBIM4 | TMEM195 | TMEM43 | TMPO | TNF | TNFRSF21 | TNNC1 | TNNI3 | TNNT2 |
| TP63 | TPM1 | TRDN | TRPM4 | TSC1 | TSEN15 | TTC21B | TTC30A | TTR | TWIST1 |
| TXNDC3 | UBE2B | UBR1 | UMODL1 | USF1 | USP34 | USP44 | VANGL2 | VCL | VEGFA |
| VEGFC | VIT | WDR5 | WHSC1 | WNT3A | XPO1 | ZEB2 | ZFPM1 | ZIC3 | ZNF480 |
| ZNF528 | ZNF534 | ZNF610 | ZNF638 | ZNHIT3 | KAT6B |  |  |  |  |

**References:**

1. Wessels, M.W. & Willems, P.J. Genetic factors in non-syndromic congenital heart malformations. *Clin Genet* **78**, 103-23 (2010).

2. Fahed, A.C., Gelb, B.D., Seidman, J.G. & Seidman, C.E. Genetics of congenital heart disease: the glass half empty. *Circ Res* **112**, 707-20 (2013).

3. Wilde, A.A. & Behr, E.R. Genetic testing for inherited cardiac disease. *Nat Rev Cardiol* **10**, 571-83 (2013).

4. Zaidi, S. *et al.* De novo mutations in histone-modifying genes in congenital heart disease. *Nature* **498**, 220-3 (2013).

5. Gelb, B.D. Recent advances in understanding the genetics of congenital heart defects. *Curr Opin Pediatr* (2013).
